# Quorum-Sensing Regulation of Antimicrobial Resistance in Bacteria

**DOI:** 10.3390/microorganisms8030425

**Published:** 2020-03-17

**Authors:** Xihong Zhao, Zixuan Yu, Tian Ding

**Affiliations:** 1 Research Center for Environmental Ecology and Engineering, Key Laboratory for Green Chemical Process of Ministry of Education, Hubei Key Laboratory of Novel Reactor and Green Chemistry Technology, School of Environmental Ecology and Biological Engineering, Wuhan Institute of Technology, Wuhan 430205, China; xhzhao2006@gmail.com (X.Z.); zixuanyu1996@163.com (Z.Y.); 2 College of Biosystems Engineering and Food Science, National-Local Joint Engineering Laboratory of Intelligent Food Technology and Equipment, Zhejiang Key Laboratory for Agro-Food Processing, Zhejiang University, Hangzhou 310058, China

**Keywords:** quorum sensing, microbial resistance, biofilm, quorum quenching

## Abstract

Quorum sensing is a cell-to-cell communication system that exists widely in the microbiome and is related to cell density. The high-density colony population can generate a sufficient number of small molecule signals, activate a variety of downstream cellular processes including virulence and drug resistance mechanisms, tolerate antibiotics, and harm the host. This article gives a general introduction to the current research status of microbial quorum-sensing systems, focuses on the role of quorum-sensing systems in regulating microbial resistance mechanisms, such as drug efflux pump and microbial biofilm formation regulation, and discusses a new strategy for the treatment of drug-resistant bacteria proposed by using quorum quenching to prevent microbial resistance.

## 1. Introduction

Quorum sensing (QS) is also called density sensing, which controls a variety of physiological behaviors in bacteria. Whether in Gram-negative or Gram-positive bacteria, quorum-sensing mechanisms exist, but the signal molecules they use to transmit information are different. Bacteria control the behavior of the entire bacterial population by synthesizing and secreting signal molecules (also known as self-inducing molecules). When the concentration of signal molecules reaches a certain threshold with the bacterial population density, the expression of certain specific genes can be started to regulate the bacterial population adaptation. The QS system regulates various cellular processes, which mainly involve the regulation of bacterial luminescence, virulence factors, disinfectants tolerance, spore formation, toxin production, motility, biofilm formation, and drug resistance. 

Antibiotics are now widely used around the world, and antibiotic resistance is spreading faster than ever before [1,2]. Since the introduction of antibiotics, the use of millions of tons of antibiotics has caused selection pressure, and almost all pathogenic bacteria have developed resistance to commonly used antibiotics [3,4,5]. Most antibiotics currently used are designed to directly kill pathogenic bacteria, such as destroying cell membranes and interfering with key protein synthesis [6]. This “life or death” selection pressure promotes the evolution of microbial resistance, and the large-scale use of antibiotics has brought serious microbial resistance issues. In recent years, “superbugs” that can resist various commonly used antibiotics have appeared around the world [7,8,9]. The increasing resistance of bacteria to antibacterial compounds and the spread of resistant pathogens have become serious threats to human health [10,11,12]. At present, most antibacterial compounds target the necessary bacterial physiological processes, thereby exerting strong selection pressure on bacteria and promoting the emergence and spread of drug-resistant strains [13]. A report commissioned by the British government recently estimated that by 2050, antimicrobial resistance could cause 10 million deaths each year and cause cumulative losses of US$ 100 trillion to world GDP [14,15]. Recently, some studies at home and abroad indicate that the QS system may be related to bacterial resistance [16,17,18]. Therefore, inhibiting bacterial QS has become a new promising antibacterial strategy, which can not only prevent the development of bacterial resistance, but also eliminate the expression of virulence factor genes related to population density.

## 2. Microbial Resistance Mechanisms

**Figure 1:**
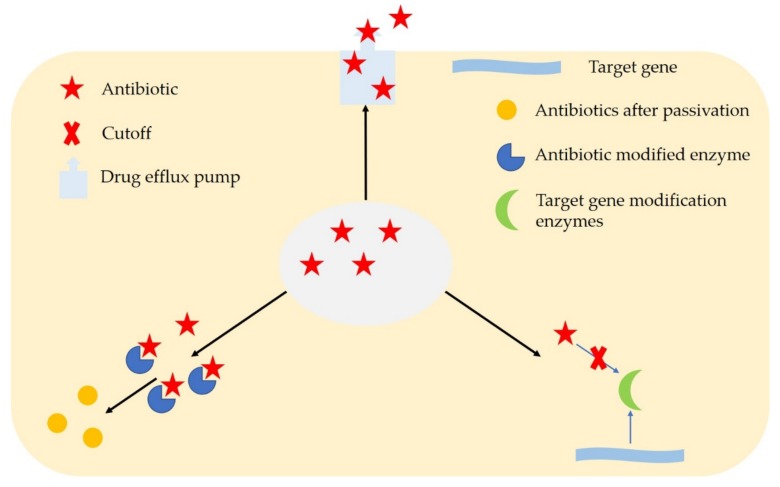
Common mechanisms of microbial resistance.

The large-scale use of antibiotics in clinical treatment has led to the formation of corresponding multi-resistance mechanisms by microorganisms against the target of antibiotics [19,20]. The main mechanisms are passivation of antibiotics through chemical modification, efflux pump systemic elimination of antibiotics, and modification of drug-targeting genes (Figure 1). At the same time, many pathogenic bacteria can form a dense biofilm [21], which makes the bacteria highly resistant. (1) Chemical modification inactivates the mechanism of antibiotic activity, that is, the secretion of a modified enzyme to change the chemical structure of antibiotic drugs, which leads to antibiotic inactivation and loss of activity. Its enzymatic mechanism includes antibiotic degradation and derivatization of antibiotic chemical groups [22]. One of the best strategies to deal with the existence of antibiotics is to produce enzymes. By adding specific chemicals to the compounds to inactivate the drugs or destroy the molecules themselves, the antibiotics cannot interact with the target substances. (2) Microorganisms can also use antibiotic pumps to expel antibiotics. Antibiotic drugs usually must enter the cells through the cell membrane of the microorganism in order to effectively attack specific targets. Antibiotic drug efflux resistance is an important mechanism of microbial resistance, which is accomplished by drug efflux pumps. Microorganisms assemble an efflux pump protein on the cell membrane to expel antibiotic drugs in the cell. The excretion rate is usually faster than the drug penetration rate, thereby controlling the drug level in the cell to a non-sensitive level. So far, there have been a variety of microbial efflux pump systems that have been discovered, such as lipophilic and hydrophilic efflux systems, which are targeted at drugs of different chemical properties [23,24]. (3) Another important resistance mechanism is the modification of drug-targeting genes. This mechanism mainly changes the drug-targeting genes through modification, which makes the drug lose its target. Another common strategy for bacteria to develop antibiotic resistance is to avoid the effects of antibiotics by interfering with the target site. As a result, bacteria have evolved different strategies, including protecting the target (avoiding the antibiotic from reaching its binding site) and modifying the target site, thereby reducing affinity for antibiotic molecules. β-lactam antibiotics play a bactericidal role by inhibiting the mucopeptide synthase, penicillin binding proteins (PBPs) of the bacterial cell wall trans peptide process, which prevents bacteria from forming a complete cell wall and dies [25]. (4) Drug resistance is due to cellular adaptation. Over the years, bacteria have developed complex mechanisms to cope with environmental stress and stress in order to survive in the harshest environments, including the human body. To gain an advantage, bacteria need to compete for nutrients and avoid attacks from molecules produced by other competing organisms [26]. In a particular host, bacteria are constantly attacked by the host’s immune system, and in order to establish themselves in a particular biological environment, it is essential that they adapt and cope with these stressful situations. Thus, bacteria have devised complex mechanisms to avoid disrupting key cellular processes, such as cell wall synthesis and membrane homeostasis. The development of resistance to daptomycin (DAP) and vancomycin (at low levels in *Staphylococcus aureus*) is the most clinically relevant example of a resistance phenotype that is the result of an overall cellular adaptive response to bacterial attack.

The existence of multiple drug resistance mechanisms has made it difficult to overcome and solve the problem of microbial resistance. In the struggle between microorganisms and antibiotics, more and more microorganisms have evolved multiple resistance mechanisms and have become “super bacteria”. For example, the super resistant bacterium *Staphylococcus aureus* can specifically degrade penicillin by producing β-lactamase, and on the other hand, it can deprive methicillin of its ability to bind cell wall mucin synthase by producing PBP2a protein [27]. In another super bacterium *Pseudomonas aeruginosa*, in addition to being able to produce different drug efflux pumps to resist multiple types of antibiotics [28,29], resistance genes can also be obtained through horizontal gene transfer [30,31], and its bacteria changes in body shape and dense biofilms can resist almost all antibiotics on the market [32,33].

In recent years, researchers have found that the formation of bacterial biofilm resistance to bacteria is particularly important. The drug resistance produced by bacterial biofilm is a systematic and complex drug resistance mechanism. The principle of drug resistance has at least the following three points [34,35,36]: (1) The biofilm itself is an effective drug barrier, which can significantly reduce it antibiotic permeability. Bacteria are connected to each other through proteins and DNA, especially extracellular polysaccharides, forming an insurmountable barrier, which can greatly reduce the permeability of antibiotic drugs and improve the survival rate of bacteria in the biofilm. (2) The special microenvironment in the biofilm makes the bacteria in the membrane produce heterogeneity and regulates the antibiotic resistance of the bacteria. The study found that the concentrations of nutrients and bacterial secretions in different areas of the biofilm were not the same, which led to the inconsistent growth status of the bacterial bodies in different areas of the biofilm, that is, the heterogeneity of the bacteria, which led to different levels of drug resistance bacterial cells. (3) The extreme environment outside the biofilm promotes drug resistance within the membrane. Some drastic environmental changes outside the biofilm, such as changes in temperature, pH, and the concentration of certain chemicals, may affect the functions of the bacteria in the biofilm to regulate its physiological and biochemical functions, reduce its growth efficiency, and form a state of resistance to antibiotics. The microbial resistance mechanisms are summarized in Table 1. Therefore, it is advantageous to study bacterial resistance through inhibiting the formation of bacterial biofilms, inhibiting the bacterial quorum, reducing the barrier effect of biofilms, and inhibiting the phenotypic changes of bacteria in biofilms to weaken the resistance of bacterial biofilms to antibiotics [37].

## 3. Microbial Quorum Sensing System and Its Regulation Mechanism

In recent years, the discovery of microbial quorum-sensing systems has provided new hope for studying the regulatory mechanism of drug resistance mechanisms and overcoming drug resistance. The results show that the quorum-sensing system regulates various cellular processes of microorganisms, such as pathogenic gene expression, toxin production and extracellular polysaccharide synthesis, and plays an important regulatory role in the process of drug efflux pumps and the formation of microbial biofilms [38,39,40]. According to different self-inducible molecules, bacterial QS systems are divided into three types. One is the QS system with acyl-homoserine lactone (AHL) as the self-inducible molecule, which exists in Gram-negative bacteria. The oligopeptides are QS systems that are self-inducing molecules and exist in Gram-positive bacteria. The other types are QS systems that use furan borate diesters as self-inducing molecules and exist in Gram-negative and Gram-positive bacteria. 

**Figure 2:**
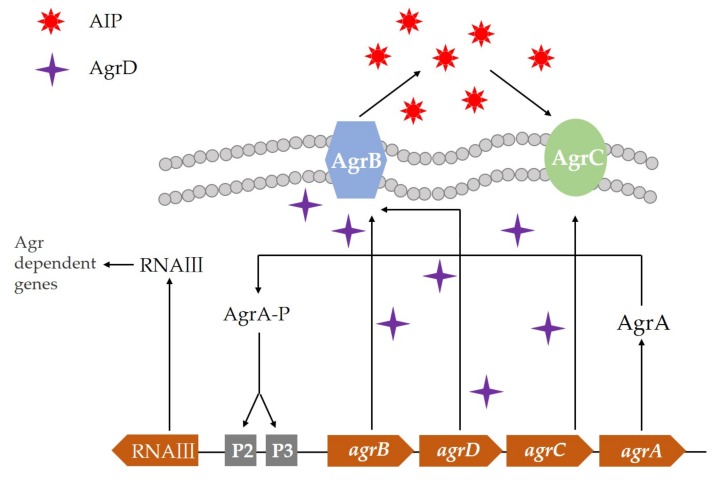
Agr quorum-sensing system in *Staphylococcus aureus*.

Signal molecules represented by small molecule oligopeptides, that is, autoinducing oligopeptide (AIP), are mainly used as quorum-sensing signal molecules in Gram-positive bacteria. AIP precursor molecules are generally formed by modification of the leader peptide, and it can be transported across the body with the help of the ATP-binding cassette (ABC) transporter for extracellular secretion. For example, note the Agr system in *Staphylococcus aureus* (Figure 2). With the increase of cell density, bacteria begin to synthesize a large number of virulence factors, thereby increasing their pathogenicity. This process is a response made by oligopeptide signal molecules to regulate gene expression and stimulate cells. When the signal molecule oligopeptide secreted to the outside reaches a certain concentration, it will bind to the receptor protein on the cell membrane and pass the phosphorylation/dephosphorylation cascade to pass the oligopeptide to the intracellular binding promoter to start the transcription mechanism and post-translational modifications that activate or inhibit the expression of the gene of interest [41].

**Figure 3:**
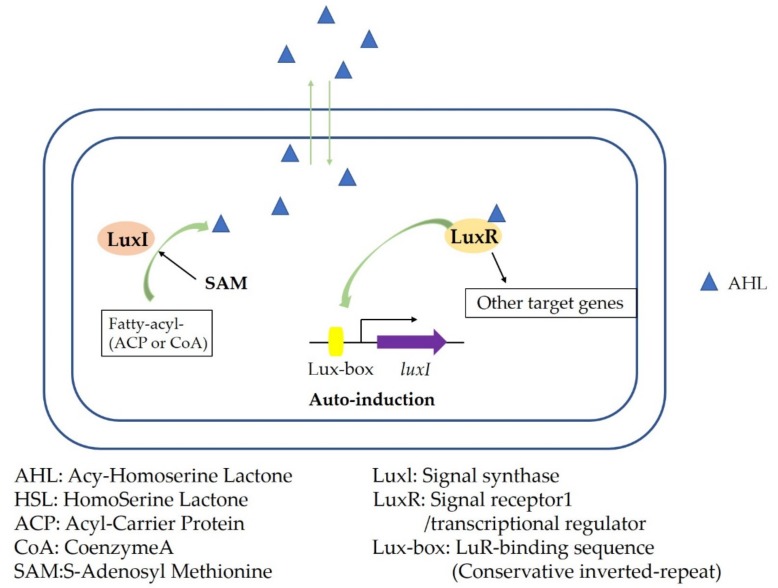
Acyl-homoserine lactone (AHL) quorum-sensing system.

The quorum-sensing signal molecules represented by acyl-homoserine lactones (AHLs), represented by autoinducer-l (AI-1), are mainly used in Gram-negative bacteria [42]. AHL, as a synthetic product in the LuxR-LuxI system, which is widely present in Gram-negative bacteria, can diffuse freely into and out of bacterial cells, and it can also accumulate in the surrounding environment (Figure 3). LuxR, which encodes the LuxR-binding protein, is a transcriptional activator that induces a response, and it can also be called a transcription regulator. Its regulation requires the activation of an acyl-AHL molecule; luxI, which encodes the LuxI protein, is an AHL synthetase. Function requires S-adenosylmethionine (SAM) and acyl-acyl carrier protein (acyl-ACP) as substrates. After AHL binds to LuxR, dimerization or multimerization occurs, and the multimerization product binds to the upstream regulatory region of the target gene to activate or inhibit the expression of the target gene [43,44,45]. The binding specificity of AHL and LuxR is very strong because the specificity of AHL is controlled by different acyl side chain groups. Therefore, Gram-negative bacteria use this method to achieve the transfer of information between cells within the species, which will avoid interference caused by external bacteria [46,47].

**Figure 4:**
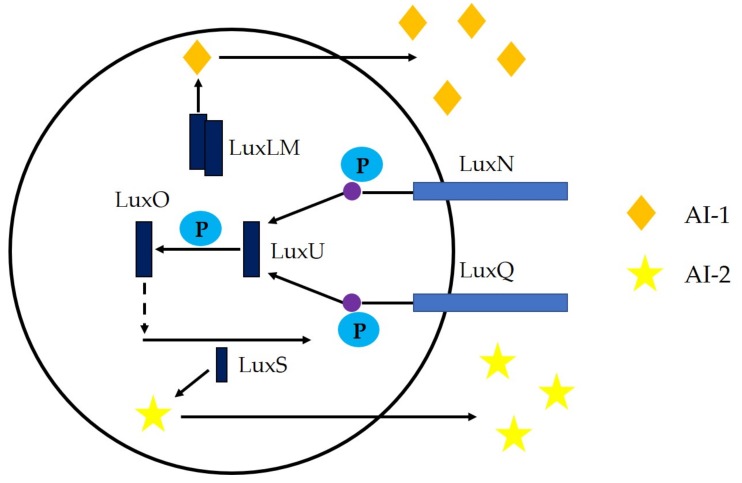
AI-2 quorum-sensing system.

The signal molecule represented by furan borate diester [48,49], which is represented by AI-2, can be used as a quorum-sensing signal molecule in both Gram-negative and Gram-positive bacteria. What is different is that AI-2 mediates the interspecies quorum-sensing system [50,51], that is, the physiological phenomenon that bacteria receive signals from AI-2 released by foreign bacteria and regulate corresponding gene expression (Figure 4). In the activated methyl cycle (AMC), AI-2 is produced from *S*-adenosylmethionine (SAM) in a three-step enzymatic reaction [52]. Among them, SAM is a methyl donor, and the intermediate product *S*-adenosylhomocysteine (SAH) is hydrolyzed by 5’-methylthioadenosine/*S*-adenosylhomocysteine ribozyme (Pfs) to *S*-ribosylhomocysteine (SRH) and adenine [53]. LuxS (S-ribosylhomocysteinase), the encoded product of *luxS*, is an AI-2 synthetase [54]. LuxS catalyzes the cleavage of SRH into DPD and homocysteine. DPD (4,5-dihydroxy-2,3-pentanedione) is a precursor substance for the synthesis of AI-2 [55,56]. The molecular structure of DPD is very unstable, and cyclization, rearrangement, and other reactions may change at any time. This shows that DPD can derive a variety of AI-2 molecules with different structures, compositions, and similarities, which can be recognized by different species of bacteria. It can also be said that Al-2 can be understood as a mixture of several similar small molecules with different conformations. There are two types of AI-2 structures that have been discovered. The molecular structure of AI-2 in *Vibrio harveyi* is a furanosyl borate diester discovered by Chen et al. [57]. Miller et al. [58] found that the molecular structure of AI-2 of *Salmonella typhimurium* is furanosyl borate diester that lacks boron.

## 4. Quorum Sensing and Biological Competition

Ecological competition refers to the process by which an organism reduces the survival or reproduction of other organisms for its own survival needs. This competition can be divided into exploitative competition and interference competition [59,60]. Exploitation competition occurs indirectly, and it manifests itself as one organism consumes another organism’s resources. Exploitation competition also occurs in microorganisms, especially when they aggregate and form dense communities (such as biofilms), mainly as nutritional restrictions strong competition for exploitation occurs between cells of the same genotype and different genotypes. Interference competition refers to competition that occurs when individuals directly harm each other. In microorganisms, it refers to the secretion of metabolites that harm other cells, including the secretion of antibiotic compounds and asphyxiating polymers [61]. Co-culture experiments have shown that these secreted factors often determine which genotypes can prevail in mixed communities [62]. Exploitation and interference competition are common in bacterial communities, which strongly affect the results of bacterial diversity [63]. At present, bacteria have evolved methods that can directly detect and respond to ecological competition. In the bacterial stress response, bacteria interact with each other and regulate a series of favorable behaviors (such as reproduction), which is called bacterial quorum sensing [64]. QS is a social trait that controls bacterial population and diversity by regulating the production of extracellular public goods, including beneficial public goods and harmful public goods [65].

The stress response to ecological competition is usually associated with the release of toxins because in bacterial community ecological competition usually has foreign genotypes and evolved genotypes [66]. Bacterial populations describe QS’s role in regulating microbial diversity through the role of toxins. Toxins produced by bacteria cause bacterial damage, and bacteria usually release toxins that kill other bacteria to break down single cells of other genotypes. Toxins are particularly useful in identifying evolutionary functions that have evolved to affect the activity of other bacteria and ultimately kill them, such as bacteriocin, a narrow-spectrum antibiotic that targets other bacteria, and pyocyanin, which has multiple potential effects on metabolism and nutrition [59]. The microbial diversity regulated by toxins is as follows: On the one hand, bacteria sense ecological competition through information related to the number of microorganisms in the community mediated by QS. The density of self-cells is a key factor in regulating the effects of toxins. QS regulation ensures that there are enough bacteria in the community with the same genotype to secrete toxins or promote growth products [67]. On the other hand, QS information can also predict the intensity of ecological competition. The diversity potential of microbial communities means that the population density information not only acts on itself, but also may be the genotype of specific signals generated by other cells, and the signal molecules are highly specific. It is possible to distinguish population density information because it can identify specific strains or species as evolutionary competitors, such as *Proteus mirabilis*. It can detect other genotype strains in the species and identify future competitors, or there are strains of quorum-receptor molecules that they do not produce. The luxR and its homologues can respond to AHLs signals of other genotypes in the community, identify competitors, and regulate biofilm formation, luminescence, release of virulence factors, swimming capacity, and expression of protease activity genes, etc., and reduce interference by interfering with competition The number of genotype strains can finally regulate the order of different genotypes in the microbial community to achieve the purpose of regulating the biodiversity in the community.

## 5. Regulation of Microbial Resistance by Quorum Sensing 

### 5.1. Regulation of Bacterial Efflux Pump by QS 

Bacterial active efflux pumps can effectively discharge antibiotics into the bacteria, and they play an important role in multidrug resistance. Bacterial active efflux pumps are generally composed of three parts: from the outside to the inside are the outer membrane channel protein, the fusion protein, and the cytoplasmic membrane efflux protein. The fusion protein connects the outer membrane channel protein and the cytoplasmic membrane efflux with the participation of energy. Protein, foreign substances including antibiotics, and their metabolites are selectively or non-selectively eliminated from bacteria. At present, QS system’s regulating effect on bacterial efflux pump has been confirmed [68,69]. The regulation effect of QS system on the expression of multidrug-resistant pumps is that both the expression of multidrug-resistant pumps can be regulated, and the QS system itself is also affected by the expression level of multidrug-resistant pumps. On the one hand, the QS system can regulate the expression of efflux pump genes. For example, *Bacteroides fragilis* ATCC25285 was cultured in vitro in the presence or absence of self-inducible molecules C_6_-HSL and C_8_-HSL. Its growth-resistant drug-sensitive efflux pump gene *bmeB* expression and the biofilm structure was tested, and it was found that LuxR, a self-inducing molecule receptor of the QS system, can respond to exogenous AHL, upregulate the expression of *bmeB* efflux pump, and form resistance to antibiotics [70]. Similarly, some researchers have found that autoinducer can up-regulate the multidrug resistance pump MexAB-OprM, causing bacteria to develop multidrug resistance [71]. On the other hand, the QS system itself is also affected by the expression level of the efflux pump. However, some researchers have found that the overexpression of the MexCD-OprJ multidrug efflux pump shuts down the QS response of *P. aeruginosa* [72]. While some efflux pumps (such as RND) expel the drug out of the cell to form drug resistance, QS system self-inducible molecules can also be expelled from the cell, increasing the concentration of extracellular self-inducible molecules, which is manifested by the exacerbation of bacterial infections [73]. This suggests that the high expression of the efflux pump may promote the further activation of the QS system, promote the QS system’s regulation of toxin infection factor synthesis and efflux pump expression, and enhance the infectivity and invasiveness of bacteria. At the same time, researchers have found that the QS system regulates biofilm-forming genes and also regulates bacterial resistance-related genes. The LuxR receptor of the QS system in *Bacteroides fragilis* has been shown to regulate biofilm formation and *bmeB* expression in efflux pumps [70]. Similarly, in the presence of imipenem, the resistance gene *ampC* can be highly expressed in biofilms [74,75]. This situation of high biofilm-forming bacteria, but not free or low-expression, suggests that the QS system may also regulate the expression of these resistance-related genes while regulating biofilm formation. This has attracted people’s attention and may be another way for the QS system to directly regulate the related genes to form drug resistance (Table 2).

### 5.2. Regulation of Bacterial Biofilm Formation by QS 

Biofilm is a special structure formed by bacteria adsorbed on the surface of inert or active materials in order to adapt to the living environment. It is composed of itself and extracellular matrix such as polysaccharides and proteins. It has a certain spatial structure and shows new biological traits and stronger environmental adaptability. Recent studies have shown that the majority of human bacterial infections are related to biofilm, and the formation of biofilm is one of the important reasons why clinical bacterial infections are difficult to cure and relapse [81]. The formation of bacterial biofilms is closely related to the development of drug resistance. It is generally believed that bacterial biofilms can lead to bacterial drug resistance through penetration restriction mechanisms, nutrition restriction mechanisms, and drug resistance phenotypic mechanisms [82]. The molecular barrier and charge barrier (mostly negatively charged) formed by the polysaccharides in the biofilm can prevent or delay the penetration of certain antibiotics, which is the main mechanism of limiting permeability [76]. The nutrient limitation mechanism is closely related to the permeation limitation mechanism. Due to the existence of the biofilm’s permeation limitation, nutrients cannot easily pass through the biofilm, which makes the nutrient in the biofilm lack nutrients and slows the growth of the inner bacteria. The slow-growing state is also called starvation, and the starvation of bacteria is less sensitive to antibiotics [36,83,84]. In addition, the study also found that the formation of drug resistance is related to the differential expression of some biofilm-related phenotype-related genes, which suggests that the formation of biofilm resistance may also have a drug-resistant phenotype mechanism [85,86,87].

In the process of biofilm formation, the QS system plays an important regulatory role. QS system regulates the biofilm formation of Gram-negative bacteria and Gram-positive bacteria. The formation of Gram-negative bacteria’s biofilm is regulated by the QS system using AHL as a signal molecule, which is composed of signal molecules and corresponding signal molecule receptors. For example, in *P. aeruginosa*, the QS system has two signaling systems: *lasl*/*lasR* and *rhlI*/*rhlI*. The *lasl*/*rhlI* and *lasR* and *rhlR* genes encode different signal molecule synthetases and signal molecule receptors, respectively [77,78]. Signal molecules increase in secretion as the density of bacteria increases. When a signal molecule reaches a certain threshold, the signal molecule binds to the corresponding signal molecule receptor and activates the receptor. The activated receptor then activates the relevant transcriptional regulators to synthesize extracellular polysaccharides, toxic factors, alginates, etc., thereby causing bacteria to form biofilms. The QS system regulates the biofilm of Gram-positive bacteria by using oligopeptides as signal molecules, which can be recognized by the two-component sensing protein after modification, and it regulates the expression of the target gene through phosphorylation and dephosphorylation of the protein. This further regulates the formation of biofilms. Different types of bacteria have different oligopeptide signaling molecules and QS system regulation pathways. For example, the two-component system in *Streptococcus* is the histidine protein kinase and response regulatory protein [88], while the QS system in *Staphylococcus aureus* is highly conserved. WalK/WalR is also called the YycG/YycF two-component system, which directly regulates the formation of biofilms [79]. In addition to two-component systems, there are also known regulatory factors that influence biofilm formation. For example, RNA nucleic acid polymerase III can promote the formation of *S. aureus* biofilms, while RNA nucleic acid polymerase III inhibits protein peptides and can significantly inhibit the production of its biofilms [80,89]. In *Streptococcus*, competent stimulating factors can promote the regulation of the biofilm by the QS system [80]. In *Staphylococcus epidermidis*, QS system-related comprehensive regulator *sarA* is closely related to biofilm formation and is a positive regulator of *Staphylococcus epidermidis* biofilm formation [90,91] (Table 2).

### 5.3. Regulation of Bacterial Secretion System by QS 

Pathogenic bacteria secrete proteins through the cell membrane. This basic process enables them to attack other microorganisms, evade the host’s immune system, cause tissue damage, and invade host cells. Secreted proteins can act as toxic factors, produce toxic substances to host cells, and may also promote adhesion to these cells. Proteins are transported on phospholipid membranes by several secretory systems [92]. The secretory system plays an important role in the spread of bacteria. So far, the structure, composition, and activity of eight secretory systems (T1, T2, T3, T4, T5, T6, T7, T9) have been determined. These differences are due to the differences between Gram-positive and Gram-negative bacteria [93,94].

The type I secretion system (T1SS) is widely distributed in Gram-negative bacteria. It has three structural elements: ABC transporter, membrane fusion protein, and outer membrane factor [95]. So far, there are two systems that can regulate the expression and secretion of T1SS substrates: the Has system of *Serratia marcescens* and *Pseudomonas aeruginosa*, and the hemolysin system of *Vibrio cholerae*, *Neisseria meningitidis*, and *E. coli* [95]. In the transcriptional study of *P. aeruginosa*, T1SS is positively regulated by QS because the expression of its effector alkaline protease AprA depends on QS [96]. The type II secretion system (T2SS) is responsible for secreting folded proteins from the periplasm in Gram-negative bacteria [92]. The main function of T2SS is to obtain nutrition. It is responsible for secreting a large amount of exoproteins. The Xcp system in *P. aeruginosa* secretes QS-regulated virulence factors elastase and exotoxin, which itself is also positively regulated by QS [97]. The QS system directly controls biofilm production related to the *Vibrio cholerae* type II secretion system [98]. The type IV secretion system is widely present in Gram-negative and Gram-positive bacteria. T4SS is the most worldwide secretion system. It can transfer not only proteins, but also DNA [99]. The QS system is directly related to T4SS in *Brucella abortus*. For the *virB* operon encoding T4SS regulated by VjbR, LuxR-type QS is responsible for the virulence characteristics of *Brucella abortus* [100].

## 6. New Strategy for Preventive Treatment of Microbial Resistance

**Figure 5:**
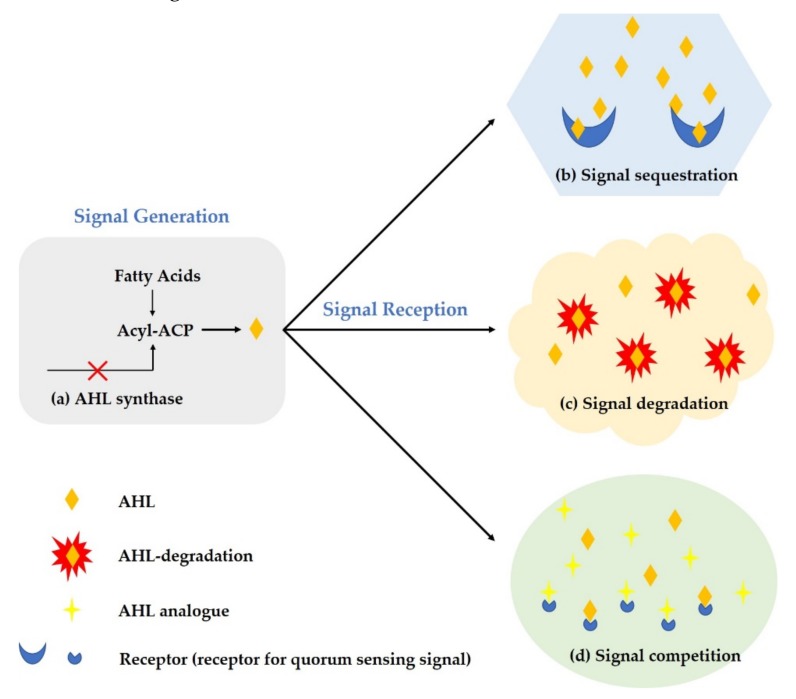
Schematic diagram of the quorum-sensing inhibitor mechanism.

The QS system plays an important role in the formation of bacterial drug resistance mechanisms by regulating the formation of biofilms and the direct regulation of drug efflux pumps. The increase in bacterial resistance has aggravated the difficulty of disease prevention, and the side effects caused by excessive use of drugs may also endanger human health. The discovery of control strategies for quorum quenching diseases in recent years has provided new possibilities for overcoming and solving the problem of microbial resistance [101,102]. By interfering with the QS system of specific microorganisms, hindering the exchange of information between microorganisms, and reducing the expression level of hazard factors, this phenomenon is called quorum quenching (QQ) [103,104]. The properties of quorum quenching molecular roles (chemical compounds, enzymes), modes of action (competition, inhibition, QS signal interdict, etc.), and targets are diverse and are all major steps in the QS pathway, including from synthesis to diffusion to accumulation, and sensing of a portion of the QS signal may be affected. Normally, the enzymes that inactivate the QS signal are called quorum-quenching enzymes, while the chemicals that disrupt the QS pathway are called QS inhibitors. The active substances with quorum quenching are collectively referred to as quorum-sensing inhibitors (QSIs). Unlike currently commonly used antibiotics, quorum-quenching agents reduce microbial infections by inhibiting microbial quorum induction, and they generally do not affect microbial growth. There are three main ways of quorum quenching: (1) inhibition of signal molecule production, (2) degradation of signal molecule, and (3) inhibition of signal molecule conduction or binding to receptors [69,105] (Figure 5). The QS system suppression strategies are summarized in Table 3. Compared with the traditional prevention and control methods, which mainly aim at inhibiting and killing microorganisms, QQ will not pressure the growth of microorganisms, nor will it induce the development of microbial resistance. Therefore, the research and development of QQ-based QSI has gradually attracted the attention of researchers and has become a new strategy for controlling harmful microorganisms.

### 6.1. Inhibition of Signal Molecule Production

In the QS system, the synthesis of signal molecules plays a vital role in the communication between cells [70]. Interfering with the synthesis of signal molecules is direct way to inhibit quorum sensing. In short, if no signal molecules are produced, quorum sensing will not occur. However, there are few studies on signal molecule synthesis inhibitors, and the data are very limited. Recombinant PS and LuxS can successfully convert SAH to homocysteine and DPD. The LuxS compound is chemically modified to generate QS signal AI-2, and its activity can be determined by monitoring the luminescence of *Vibrio harvey BB170*. The researchers screened peptides obtained after three rounds of selection to inhibit LuxS enzyme activity. The corresponding synthetic peptide TNRHNPHHLHHV showed a specific inhibitory effect on LuxS [106]. Schramm et al. synthesized MT-DADMe-ImmA, a picomolar inhibitor, through 5′-methylthioadenosine phosphorylase (MTAP) transition state structure [107]. It blocks QS in *Vibrio cholerae* without affecting the growth rate of the bacteria.

The compounds based on (2-nitrophenyl) methanol are considered promising PqsD inhibitors. PqsD is a key enzyme for signal molecule biosynthesis in *Pseudomonas aeruginosa* in intercellular communication. Studies have found that (2-nitrophenyl) methanol derivatives have improved cell efficacy and provide new prospects for the application of PqsD inhibitors as anti-infective drugs [108]. A study revealed a well-proven but underdeveloped novel inhibitor of the target enoyl-ACP reductase that promotes the acyl chain length of N-acyl homoserine lactones, of which an ester is the main signaling molecule in Gram-negative bacteria [109].

### 6.2. Degradation of Signal Molecule

Degradation of signal molecules is a more studied quenching method. This method mainly uses quorum-quenching enzymes produced by microorganisms or other organisms to degrade quorum-sensing signal molecules, so that the concentration of signal molecules is lower than the threshold, and pathogenic bacteria cannot express pathogenic genes and produce pathogenic factors, thus losing the ability to infect the host. 

Dong et al. [103] showed that the aiiA gene encoding the AHL degrading enzyme in *Bacillus* sp. 240B1 was expressed in potato and tobacco. The expression of aiiA gene in transgenic tobacco and potato significantly increased resistance to *Pseudomonas legume*. After inoculation with pathogenic bacteria, potato tubers and tobacco leaves did not present with disease spots nor was the appearance of disease spots significantly delayed, which proved that enzymatic degradation of AHL signal molecules is indeed an effective disease prevention and control method. The quorum-sensing system using AHLs as a signal for many pathogenic bacteria is an important regulator of virulence and an attractive target for anti-infective drugs. This method of prevention and control does not act on the pathogenic bacteria itself, but it acts on the signal molecules produced by the pathogenic bacteria so that the pathogenic genes are not expressed. Thus, the “life or death” selection pressure is not exerted on the pathogenic bacteria, and the possibility of bacteria becoming resistant and causing disease is greatly reduced, which is favorable for use as a highly effective and long-lasting drug [123,124,125,126]. Researchers identified an AidB from a soil bacterium, *Bosea sp.* strain *F3-2* [110]. It is a new AHL lactonase that hydrolyzes the ester bonds of the homoserine lactone (HSL) ring. The expression of AidB reduced the AHL signal and the production of QS-dependent virulence factors by *Pseudomonas aeruginosa* and *Pectobacterium carotovorum.* It is worth noting that AidB is a thermostable enzyme, which retains its catalytic activity after being treated at 80 °C for 30 min, and it shows reliable storage stability at 4 °C and room temperature. These characteristics may make it more suitable for practical applications. Zhang et al. [111] connected the target fragment MomL with pNCMO2 to obtain a recombinant strain named *Bb*MomL. The *Bb*MomL can not only degrade the exogenous signal molecule C6-HSL, but it also degrades AHL signal molecules produced by the Gram-negative pathogen botulinum. Compared to wild-type *Bifidobacterium breve*, *Bb*MomL not only inhibits fungal and Gram-positive bacterial pathogens, but it also significantly inhibits Gram-negative bacterial pathogens. The results show that *Bb*MomL has a wide antibacterial spectrum. In addition, the *Bb*MomL significantly reduced the secretion of pathogenic factors and the pathogenicity of *Pseudomonas aeruginosa*. Research by Cui et al. showed that crude extracts from *Lactobacillus crustorum ZHG 2-1* can degrade AHL [112].

Signal molecule inactivation or denaturation can be achieved through a variety of mechanisms, which is the most basic approach to using QS to prevent bacterial resistance and to study new antibacterial strategies [127]. Some microorganisms can metabolize AI-2, thereby inhibiting the function of QS. The addition of ATP and LsrK (AI-2 kinase) in bacterial culture can achieve the degradation of signal molecules, and AI-2 is phosphorylated outside the cell; bacterial crosstalk controlled by AI-2 is significantly reduced [128]. AI-2 molecules are more hydrophilic after phosphorylation and are thought to be unable to pass through the cell membrane and serve as QS signals [129]. AI-2 molecules that are phosphorylated in vitro prevented the QS response of *E. coli*, *Haber’s bacillus*, and *Salmonella typhimurium* [114]. This strategy may be useful in mixed infections because LsrK can phosphorylate DPD as a precursor molecule of AI-2 [130]. In addition, it may be effective regardless of the structure and transport/sensor mechanism of AI-2 for different bacterial QS systems [131]. It has been reported that exogenous imidazole is a furan ring analog of AI-2, which reduces the resistance of *E. coli* to β-lactam antibiotics by inhibiting the function of AI-2 [113].

### 6.3. Inhibition of Signal Molecule Conduction or Binding to Receptors

In addition, quorum-inducing signal inhibitors also play an important role in reducing the pathogenicity of bacteria. Studies have found that many organisms can secrete quorum-sensing signal analogs, competitively combine with bacterial quorum-sensing signal receptors, interfere with the regulation of the quorum-sensing system, and significantly reduce the pathogenicity of bacteria. Wei et al. [115] discovered a medicinal herb extract (MHE). The *Pseudomonas* quinolone signaling (PQS) system was completely suppressed, the *rhlR/rhlI* QS system was moderately suppressed, and the *lasR/lasI* QS system was only slightly affected, suggesting that MHE may selectively target the PQS system to inhibit bacterial toxicity. In addition, electrophoretic mobility shift assays showed that MHE inhibited the binding of MvfR to the corresponding pqsA promoter region, suggesting that MHE acts as a competitor to quench the QS function in *P. aeruginosa*. Truchado et al. [116] found that flavonoids rich in *Citrus sinensis* have the function of inhibiting quorum-sensing signals, which can significantly reduce the concentration of quorum-sensing signals secreted by *Yersinia enterocolitica* and the formation of quorum-sensed biofilms without affecting bacterial growth. 

The results showed that D-galactose inhibited AI-2 activity, which could inhibit periodontal pathogens from forming biofilms [117]. The D-galactose-binding protein is highly similar to ribose-binding protein (RbsB), which is the AI-2 receptor for *Actinomycetes*. A small peptide 5906 was identified, which inhibited the LuxS activity of *Edwardsiella tarda* by specifically binding LuxS in a manner that might prevent the formation of functionally identical LuxS dimers [118]. The QS response mediated by AI-2 usually occurs in a bacterial community composed of different types of microorganisms. Multiple studies have shown that the QS phenotype in a variety of microbial communities mediates activity between normal flora and pathogens [43,132]. It is precisely because LuxS/AI-2 regulates pathogen virulence in a variety of microbial communications that disrupting and preventing signaling in these networks provides an excellent target for QS quenchers [69,133,134].

Although quorum-sensing signal antagonistic activity has been found in many bacterial, fungal, plant, and animal metabolites, only a few active molecules have been isolated and identified. Therefore, many researchers have used artificial synthesis to synthesize quorum-sensing signal analogs to antagonize the quorum-sensing signal of pathogenic bacteria and have achieved certain results [135]. Five different haloquinone analogs were tested carrying different positions of methoxy and hydroxyl [119]. Tests of Wnt activity in cell culture and *Xenopus* embryos have shown that two of these compounds can be effective inhibitors of abnormally activated Wnt/β-catenin signaling. Researchers have resolved a 2*H*-pyran-3(6H)-one derivative to develop a new asymmetric catalytic method for synthesizing collections of compounds inspired by iridescent compounds [120]. The desired product is efficiently formed with high diastereoselectivity and enantioselectivity. Evaluation of the obtained compound set led to the discovery of novel Wnt and Hedgehog signaling pathway inhibitors. Smith et al. [136] chemically synthesized a series of structural analogues of LasR, a receptor protein of the *Pseudomonas aeruginosa* quorum sensing system, and subsequent experiments showed that this series of analogues can produce antagonistic effects and inhibit the expression of virulence factors of pathogenic bacteria. In one study, researchers synthesized a group of six compounds based on a scaffold (alkylquinoxaline-2(1H)-one), which is a new anti-QS feature of the *Aeromonas caviae* Sch3 [121]. This preliminary study will help develop anti-QS compounds to overcome the clinical challenges of drug-resistant strains. In addition, some researchers have synthesized known QS inhibitors designed on the N-(3-oxododecanoyl) homoserine lactone (3O-C12-HSL) QS molecular scaffold [122]. The LasR antagonist interacts with the N-terminal ligand binding domain of LasR, thereby blocking the binding site of the QS molecule. They were coupled with ciprofloxacin to inhibit the formation of *P. aeruginosa* biofilms and increase the antibiotic sensitivity of clinical strains.

As mentioned earlier, in addition to affecting the expression of pathogenic genes, the quorum-sensing system also plays an important role in regulating biofilm formation and overexpression of drug efflux pumps. Therefore, in addition to being used directly for the prevention and control of microbial diseases, population quenchers can also be used simultaneously with antibiotics to reduce the resistance of microorganisms and increase the bactericidal efficacy of antibiotics.

## 7. Future Outlook

Quorum sensing, as an emerging research area in microbial research, has gradually received attention since the 1990s. At present, many related studies are still incomplete, and the field of drug resistance is relatively late in the field of quorum sensing. A lot of fruitful work has been done, but the related regulatory mechanism of quorum sensing in microbial resistance is still unclear. It can be seen that future research on quorum sensing in the field of microbial resistance is full of opportunities and challenges. In order to better adapt to this development, future research on quorum sensing in the field of microbial resistance should focus on the following areas: (1) In view of the incompleteness of the current QS-related regulatory mechanisms, related research should be further improved by means of molecular biology. (2) In view of the complexity of the microbial drug resistance system, we should focus on strengthening relevant research on bacterial quorum sensing. (3) In view of the inefficiency of current QSI screening, it is important to focus on establishing new and efficient QSI screening technologies. (4) In view of the broad prospects of QS systems in synthetic biology, research and discovery of multiple QS regulation components should focus on rigorous regulation of target genes to meet the diversity of engineering bacteria in actual production.

The diversity of microbial resistance mechanisms has undoubtedly brought great challenges to research and solve the problem of resistance. Therefore, it is of great practical significance to study the formation and regulation of the main resistance mechanisms of microorganisms for the prevention and control of microbial diseases. At present, research on the regulation mechanism of microbial resistance is scarce. Although studies have shown that, in *Pseudomonas aeruginosa* and several other pathogens, the quorum-sensing system is involved in the regulation of biofilm formation and drug efflux pump gene expression, the formation and regulation of drug resistance mechanisms of more clinically important pathogens still need further study. Therefore, advances and breakthroughs in research on quorum sensing and other regulatory systems will likely inject new vitality into the study of microbial resistance.

The study of microbial quorum sensing has its complexities. At the same time, research on the relationship between quorum sensing and drug resistance also faces many challenges. The quorum-sensing mechanism in many microorganisms is not singular. If the same phenotype of bacteria is regulated by multiple signaling networks, will the inhibition of one signaling mechanism be replaced by other communication mechanisms? Will inhibition of one communication mechanism cause other previously disabled regulatory mechanisms to be disabled? Recovery is a problem that may be faced in future scientific research. Finding quorum-sensing regulatory systems that are critical to microbial pathogenicity and drug resistance will be the focus of future research.

## 8. Conclusions

Although there are still many shortcomings in research on QS, with continuous maturation of molecular biology, synthetic biology, and omics, future research on QS will definitely enter a new stage, and this will also make new contributions to the development of the microbial resistance industry.

## Figures and Tables

**Table 1:** Microbial resistance mechanisms.

Resistance Mechanism	Action Mechanism
Chemical modification	Change the chemical structure of antibiotic drugs
Efflux pump system	Discharge intracellular antibiotic drugs
Modification of drug-targeting genes	Change drug-targeting genes
Global cellular adaptation	Adapt and cope with stress
Biofilm itself	Reduce the permeability of antibiotics
Special internal environment in biofilm	Intracellular thallus produce heterogeneity
Extreme environment outside the biofilm	Intracellular thallus produce resistance

**Table 2:** Regulation of microbial resistance by quorum sensing.

Regulation Type	Regulation Method	Biological Effect	Reference
**Bacterial active efflux pumps**			
Autoinducer C_6_-HSL and C_8_-HSL	QS regulate the expression of efflux pump genes	Upregulate the expression of *bmeB* efflux pump	[70]
Quorum-sensing autoinducer C_4_-HSL	QS regulate the expression of efflux pump genes	Upregulate resistance pump MexAB-OprM	[71]
4-hydroxy-2-heptylquinoline excretion	QS affected by the expression level of efflux pump	Shut down the QS response	[72]
RND efflux pump	Self-inducible molecules excretion	Exacerbate bacterial infections	[73]
**Biofilm**			
Polysaccharides	Form molecular barrier and charge barrier	Prevent or delay the penetration of antibiotics	[76]
Permeation limitation	Lack of nutrients	Less sensitive to antibiotics	[36]
*lasl/lasR* and *rhlI/rhlI* signaling system	Activate related transcription regulators	Form biofilms	[77]
Interspecies signal analogues	Regulate or inhibit enzyme activity	Altered biofilm formation	[78]
WalK/WalR two-component system		Directly regulates biofilm formation	[79]
Stimulating factors	Promote QS system adjustment	Regulate biofilm formation	[80]

**Table 3:** Review of QS system inhibition strategies.

Quenching Way	Function Method	Biological Effect	Reference
**Inhibition of signal molecule production**			
TNRHNPHHLHHV (peptide)	Inhibit LuxS enzyme activity	Inhibit AI-2 production	[106]
MT-DADMe-ImmA	Picomolar inhibitor	Inhibit AI-2 production	[107]
(2-nitrophenyl) methanol derivatives	Inhibit enzyme of signal molecule biosynthesis	Inhibit the production of signaling molecules	[108]
FabI derivatives	Inhibit enzyme activity	Inhibit the production of signaling molecules	[109]
**Degradation of signal molecule**			
The *aiiA* gene in *Bacillus sp. 240B1*	The *aiiA* gene encodes AHL degrading enzyme	Enzymes degrade AHL signaling molecules	[103]
Thermostable AHL lactonase (AidB)	Hydrolyzing the ester bond of the HSL ring	Degrade AHL	[110]
A recombinant strain, named *Bb*MomL	MomL causes loss of AHL	Degrade AHL	[111]
Crude extracts from *Lb. crustorum* ZHG 2-1	Crude extract can degrade AHL	Degrade AHL by 37.1–87.6% at 3 sub-MICs	[112]
Imidazole	Degrade AI-2	Inhibit AI-2 function	[113]
Externally added addition of ATP and LsrK	Phosphorylation and degradation of AI-2	Reduced QS reaction	[114]
**Inhibition of signal molecule conduction or binding to receptors**			
Medicinal herb extracts (MHE)	MHE as a competitive agent	Inhibit QS system	[115]
Flavonoids compounds	Reduce QS signal concentration	Inhibit QS signal	[116]
D-galactose	As an inhibitor of AI-2 activity	Inhibit AI-2 activity	[117]
A small peptide 5906	Prevents homodimer formation	Inhibit LuxS activity	[118]
Haloquinone analogs	Block endogenous Wnt-driven transcription	Inhibit Wnt/β-Catenin signaling	[119]
2H-pyran-3(6H)-one derivatives	As an inhibitor	Inhibit signaling pathway	[120]
Alkyl-Quinoxalin-2(1H)-One derivatives	The QS inhibition of these compounds	Reduce or inhibit biofilm formation	[121]
N-(3-oxododecanoyl) homoserine lactone derivatives	Block the binding site of the QS molecule	Inhibit the formation of biofilms and increase the antibiotic sensitivity	[122]

## References

[1] Chioro A., Coll-Seck A.M., Hoie B., Moeloek N., Motsoaledi A., Rajatanavin R., Touraine M. (2015). Antimicrobial resistance: A priority for global health action. Bull. World Health Organ..

[2] Abdula N., Macharia J., Motsoaledi A., Swaminathan S., Vijay Raghavan K. (2016). National action for global gains in antimicrobial resistance. Lancet.

[3] Laxminarayan R., Duse A., Wattal C., Zaidi A.K.M., Wertheim H.F.L., Sumpradit N., Vlieghe E., Levy Hara G., Gould I.M., Goossens H. (2013). Antibiotic resistance-the need for global solutions. Lancet Infect. Dis..

[4] Zhao X., Zhao F., Wang J., Zhong N. (2017). Biofilm formation and control strategies of foodborne pathogens: Food safety perspectives. RSC Adv..

[5] Liao X., Ma Y., Daliri E.B.-M., Koseki S., Wei S., Liu D., Ye X., Chen S., Ding T. (2019). Interplay of antibiotic resistance and food-associated stress tolerance in foodborne pathogens. Trends Food Sci. Technol..

[6] Nikaido H. (2009). Multidrug Resistance in Bacteria. Annu. Rev. Biochem..

[7] Tacconelli E., Carrara E., Savoldi A., Harbarth S., Mendelson M., Monnet D.L., Pulcini C., Kahlmeter G., Kluytmans J., Carmeli Y. (2018). Discovery, research, and development of new antibiotics: The WHO priority list of antibiotic-resistant bacteria and tuberculosis. Lancet Infect. Dis..

[8] Cassini A., Hogberg L.D., Plachouras D., Quattrocchi A., Hoxha A., Simonsen G.S., Colomb-Cotinat M., Kretzschmar M.E., Devleesschauwer B., Cecchini M. (2019). Attributable deaths and disability-adjusted life-years caused by infections with antibiotic-resistant bacteria in the EU and the European Economic Area in 2015: A population-level modelling analysis. Lancet Infect. Dis..

[9] Sharma V.K., Johnson N., Cizmas L., McDonald T.J., Kim H. (2016). A review of the influence of treatment strategies on antibiotic resistant bacteria and antibiotic resistance genes. Chemosphere.

[10] Zhong J., Zhao X. (2018). Isothermal Amplification Technologies for the Detection of Foodborne Pathogens. Food Anal. Meth..

[11] Ma Y., Lan G., Li C., Cambaza E.M., Liu D., Ye X., Chen S., Ding T. (2019). Stress tolerance of Staphylococcus aureus with different antibiotic resistance profiles. Microb. Pathog..

[12] Li J., Liu D.H., Tian X.J., Koseki S., Chen S.G., Ye X.Q., Ding T. (2019). Novel antibacterial modalities against methicillin resistant Staphylococcus aureus derived from plants. Crit. Rev. Food Sci. Nutr..

[13] Munguia J., Nizet V. (2017). Pharmacological Targeting of the Host-Pathogen Interaction: Alternatives to Classical Antibiotics to Combat Drug-Resistant Superbugs. Trends Pharmacol. Sci..

[14] O’Niel J. (2016). Tackling Drug-Resistant Infections Globally: Final Report and Recommendations.

[15] Whiteley M., Diggle S.P., Greenberg E.P. (2017). Progress in and promise of bacterial quorum sensing research. Nature.

[16] Haque S., Ahmad F., Dar S.A., Jawed A., Mandal R.K., Wahid M., Lohani M., Khan S., Singh V., Akhter N. (2018). Developments in strategies for Quorum Sensing virulence factor inhibition to combat bacterial drug resistance. Microb. Pathog..

[17] Jiang Q., Chen J., Yang C., Yin Y., Yao K. (2019). Quorum Sensing: A Prospective Therapeutic Target for Bacterial Diseases. BioMed Res. Int..

[18] Zhao X.H., Zhong J.L., Wei C.J., Lin C.W., Ding T. (2017). Current Perspectives on Viable but Non-culturable State in Foodborne Pathogens. Front. Microbiol..

[19] Munita J.M., Arias C.A. (2016). Mechanisms of Antibiotic Resistance. Microbiol. Spectr..

[20] Zhao X.H., Zhao F.H., Zhong N.J. (2020). Production of diacylglycerols through glycerolysis with SBA-15 supported Thermomyces lanuginosus lipase as catalyst. J. Sci. Food Agric..

[21] Rajput A., Thakur A., Sharma S., Kumar M. (2018). aBiofilm: A resource of anti-biofilm agents and their potential implications in targeting antibiotic drug resistance. Nucleic Acids Res..

[22] Liu L.Y., Ye C.X., Soteyome T., Zhao X.H., Xia J., Xu W.Y., Mao Y.Z., Peng R.X., Chen J.X., Xu Z.B. (2019). Inhibitory effects of two types of food additives on biofilm formation by foodborne pathogens. MicrobiologyOpen.

[23] Kumar S., Varela M.F. (2012). Biochemistry of Bacterial Multidrug Efflux Pumps. Int. J. Mol. Sci..

[24] Wasaznik A., Grinholc M., Bielawski K.P. (2009). Active efflux as the multidrug resistance mechanism. Postepy Hig. I Med. Dosw..

[25] Sauvage E., Terrak M. (2016). Glycosyltransferases and Transpeptidases/Penicillin-Binding Proteins: Valuable Targets for New Antibacterials. Antibiotics.

[26] Zhao X.H., Xia J., Liu Y. (2019). Contrast of Real-Time Fluorescent PCR Methods for Detection of Escherichia coli O157:H7 and of Introducing an Internal Amplification Control. Microorganisms.

[27] Peacock S.J., Paterson G.K. (2015). Mechanisms of Methicillin Resistance in Staphylococcus aureus. Annu. Rev. Biochem..

[28] Shriram V., Khare T., Bhagwat R., Shukla R., Kumar V. (2018). Inhibiting Bacterial Drug Efflux Pumps via Phyto-Therapeutics to Combat Threatening Antimicrobial Resistance. Front. Microbiol..

[29] Poole K. (2004). Efflux-mediated multiresistance in Gram-negative bacteria. Clin. Microbiol. Infect..

[30] De la Cruz F., Davies J. (2000). Horizontal gene transfer and the origin of species: Lessons from bacteria. Trends Microbiol..

[31] Luis Martinez J. (2018). Ecology and Evolution of Chromosomal Gene Transfer between Environmental Microorganisms and Pathogens. Microbiol. Spectr..

[32] Mah T.-F., Pitts B., Pellock B., Walker G.C., Stewart P.S., O’Toole G.A. (2003). A genetic basis for Pseudomonas aeruginosa biofilm antibiotic resistance. Nature.

[33] Yan S.M., Wu G. (2019). Can Biofilm Be Reversed Through Quorum Sensing in Pseudomonas aeruginosa?. Front. Microbiol..

[34] Stewart P.S. (2002). Mechanisms of antibiotic resistance in bacterial biofilms. Int. J. Med. Microbiol..

[35] Balcazar J.L., Subirats J., Borrego C.M. (2015). The role of biofilms as environmental reservoirs of antibiotic resistance. Front. Microbiol..

[36] Hathroubi S., Mekni M.A., Domenico P., Dao N., Jacques M. (2017). Biofilms: Microbial Shelters Against Antibiotics. Microb. Drug Resist..

[37] Zhong J., Zhao X. (2019). Transcriptomic Analysis of Viable but Non-Culturable Escherichia coli O157:H7 Formation Induced by Low Temperature. Microorganisms.

[38] Bauerle T., Fischer A., Speck T., Bechinger C. (2018). Self-organization of active particles by quorum sensing rules. Nat. Commun..

[39] Turan N.B., Chormey D.S., Buyukpinar C., Engin G.O., Bakirdere S. (2017). Quorum sensing: Little talks for an effective bacterial coordination. Trac-Trends Anal. Chem..

[40] Wei C.J., Zhao X.H. (2018). Induction of Viable but Nonculturable Escherichia coli O157:H7 by Low Temperature and Its Resuscitation. Front. Microbiol..

[41] Monnet V., Gardan R. (2015). Quorum-sensing regulators in Gram-positive bacteria: “cherchez le peptide”. Mol. Microbiol..

[42] Zhang W.W., Li C.H. (2016). Exploiting Quorum Sensing Interfering Strategies in Gram-Negative Bacteria for the Enhancement of Environmental Applications. Front. Microbiol..

[43] Papenfort K., Bassler B.L. (2016). Quorum sensing signal-response systems in Gram-negative bacteria. Nat. Rev. Microbiol..

[44] Chen Z., Xiang J. (2016). Advances in the research of LuxR family protein in quorum-sensing system of gram-negative bacteria. Chin. J. Burn..

[45] Banerjee G., Ray A.K. (2016). The talking language in some major Gram-negative bacteria. Arch. Microbiol..

[46] Li X., Zhang G., Zhu Y., Bi J., Hao H., Hou H. (2019). Effect of the luxI/R gene on AHL-signaling molecules and QS regulatory mechanism in Hafnia alvei H4. Amb Express.

[47] Ng Y.K., Grasso M., Wright V., Garcia V., Williams P., Atkinson S. (2018). The Quorum Sensing System of Yersinia enterocolitica 8081 Regulates Swimming Motility, Host Cell Attachment, and Virulence Plasmid Maintenance. Genes.

[48] Zhao J., Quan C., Jin L., Chen M. (2018). Production, detection and application perspectives of quorum sensing autoinducer-2 in bacteria. J. Biotechnol..

[49] Park H., Lee K., Yeo S., Shin H., Holzapfel W.H. (2017). Autoinducer-2 Quorum Sensing Influences Viability of Escherichia coli O157:H7 under Osmotic and In Vitro Gastrointestinal Stress Conditions. Front. Microbiol..

[50] Pereira C.S., Thompson J.A., Xavier K.B. (2013). AI-2-mediated signalling in bacteria. Fems Microbiol. Rev..

[51] Xavier K.B. (2018). Bacterial interspecies quorum sensing in the mammalian gut microbiota. C. R. Biol..

[52] Wang Q., He Z.Y., Hu Y.J., Jiang Y.T., Ma R., Tang Z.S., Liang J.P., Liu Z., Huang Z.W. (2012). luxS Mutant Regulation: Quorum Sensing Impairment or Methylation Disorder?. Sensors.

[53] Vendeville A., Winzer K., Heurlier K., Tang C.M., Hardie K.R. (2005). Making ‘sense’ of metabolism: Autoinducer-2, LuxS and pathogenic bacteria. Nat. Rev. Microbiol..

[54] Xue T., Yu L.M., Shang F., Li W.C., Zhang M., Ni J.T., Chen X.L. (2016). Short communication: The role of autoinducer 2 (AI-2) on antibiotic resistance regulation in an Escherichia coli strain isolated from a dairy cow with mastitis. J. Dairy Sci..

[55] Frezza M., Soulere L., Balestrino D., Gohar M., Deshayes C., Queneau Y., Forestier C., Doutheau A. (2007). Ac2-DPD, the bis-(O)-acetylated derivative of 4,5-dihydroxy-2,3-pentanedione (DPD) is a convenient stable precursor of bacterial quorum sensing autoinducer AI-2. Bioorg. Med. Chem. Lett..

[56] Stotani S., Gatta V., Medda F., Padmanaban M., Karawajczyk A., Tammela P., Giordanetto F., Tzalis D., Collina S. (2018). A Versatile Strategy for the Synthesis of 4,5-Dihydroxy-2,3-Pentanedione (DPD) and Related Compounds as Potential Modulators of Bacterial Quorum Sensing. Molecules.

[57] Chen X., Schauder S., Potier N., Van Dorsselaer A., Pelczer I., Bassler B.L., Hughson F.M. (2002). Structural identification of a bacterial quorum-sensing signal containing boron. Nature.

[58] Miller S.T., Xavier K.B., Campagna S.R., Taga M.E., Semmelhack M.F., Bassler B.L., Hughson F.M. (2004). Salmonella typhimurium recognizes a chemically distinct form of the bacterial quorum-sensing signal AI-2. Mol. Cell.

[59] Cornforth D.M., Foster K.R. (2013). Competition sensing: The social side of bacterial stress responses. Nat. Rev. Microbiol..

[60] Stubbendieck R.M., Straight P.D. (2016). Multifaceted interfaces of bacterial competition. J. Bacteriol..

[61] Stubbendieck R.M., Vargas-Bautista C., Straight P.D. (2016). Bacterial communities: Interactions to scale. Front. Microbiol..

[62] Nadell C.D., Bassler B.L. (2011). A fitness trade-off between local competition and dispersal in Vibrio cholerae biofilms. Proc. Natl. Acad. Sci. USA.

[63] Khare A., Tavazoie S. (2015). Multifactorial competition and resistance in a two-species bacterial system. PLoS Genet..

[64] Li Y.H., Tian X.L. (2016). Quorum sensing and bacterial social interactions in biofilms: Bacterial cooperation and competition. Stress Environ. Regul. Gene Expr. Adapt. Bact..

[65] Evans K.C., Benomar S., Camuy-Vélez L.A., Nasseri E.B., Wang X., Neuenswander B., Chandler J.R. (2018). Quorum-sensing control of antibiotic resistance stabilizes cooperation in Chromobacterium violaceum. Isme J..

[66] Poole K. (2012). Bacterial stress responses as determinants of antimicrobial resistance. J. Antimicrob. Chemother..

[67] Doekes H.M., De Boer R.J., Hermsen R. (2019). Toxin production spontaneously becomes regulated by local cell density in evolving bacterial populations. Plos Comput. Biol..

[68] Subhadra B., Oh M.H., Choi C.H. (2019). RND efflux pump systems in Acinetobacter, with special emphasis on their role in quorum sensing. J. Bacteriol. Virol..

[69] Wang Y., Liu B., Grenier D., Yi L. (2019). Regulatory Mechanisms of the LuxS/AI-2 System and Bacterial Resistance. Antimicrob. Agents Chemother..

[70] Pumbwe L., Skilbeck C.A., Wexler H.M. (2008). Presence of quorum-sensing systems associated with multidrug resistance and biofilm formation in Bacteroides fragilis. Microb. Ecol..

[71] Maseda H., Sawada I., Saito K., Uchiyama H., Nakae T., Nomura N. (2004). Enhancement of the mexAB-oprM efflux pump expression by a quorum-sensing autoinducer and its cancellation by a regulator, MexT, of the mexEF-oprN efflux pump operon in Pseudomonas aeruginosa. Antimicrob. Agents Chemother..

[72] Alcalde-Rico M., Olivares-Pacheco J., Alvarez-Ortega C., Camara M., Martinez J.L. (2018). Role of the Multidrug Resistance Efflux Pump MexCD-OprJ in the Pseudomonas aeruginosa Quorum Sensing Response. Front. Microbiol..

[73] Li X.Z., Plesiat P., Nikaido H. (2015). The Challenge of Efflux-Mediated Antibiotic Resistance in Gram-Negative Bacteria. Clin. Microbiol. Rev..

[74] Bagge N., Schuster M., Hentzer M., Ciofu O., Givskov M., Greenberg E.P., Hoiby N. (2004). Pseudomonas aeruginosa biofilms exposed to imipenem exhibit changes in global gene expression and beta-lactamase and alginate production. Antimicrob. Agents Chemother..

[75] Dhabaan G.N., AbuBakar S., Cerqueira G.M., Al-Haroni M., Pang S.P., Hassan H. (2016). Imipenem Treatment Induces Expression of Important Genes and Phenotypes in a Resistant Acinetobacter baumannii Isolate. Antimicrob. Agents Chemother..

[76] Roy R., Tiwari M., Donelli G., Tiwari V. (2018). Strategies for combating bacterial biofilms: A focus on anti-biofilm agents and their mechanisms of action. Virulence.

[77] Williams P., Camara M. (2009). Quorum sensing and environmental adaptation in Pseudomonas aeruginosa: A tale of regulatory networks and multifunctional signal molecules. Curr. Opin. Microbiol..

[78] An S.Q., Murtagh J., Twomey K.B., Gupta M.K., O’Sullivan T.P., Ingram R., Valvano M.A., Tang J.L. (2019). Modulation of antibiotic sensitivity and biofilm formation in Pseudomonas aeruginosa by interspecies signal analogues. Nat. Commun..

[79] Dubrac S., Boneca I.G., Poupel O., Msadek T. (2007). New insights into the WalK/WalR (YycG/YycF) essential signal transduction pathway reveal a major role in controlling cell wall metabolism and biofilm formation in Staphylococcus aureus. J. Bacteriol..

[80] Tan L., Li S.R., Jiang B., Hu X.M., Li S. (2018). Therapeutic Targeting of the Staphylococcus aureus Accessory Gene Regulator (agr) System. Front. Microbiol..

[81] France M.T., Cornea A., Kehlet-Delgado H., Forney L.J. (2019). Spatial structure facilitates the accumulation and persistence of antibiotic-resistant mutants in biofilms. Evol. Appl..

[82] Saxena P., Joshi Y., Rawat K., Bisht R. (2019). Biofilms: Architecture, Resistance, Quorum Sensing and Control Mechanisms. Indian J. Microbiol..

[83] Chebotar I.V., Mayansky А.N., Konchakova Е.D., Lazareva А.V., Chistyakova V.P. (2012). Antimicrobial Resistance of Bacteria in Biofilms. Klin. Mikrobiol. I Antimikrobn. Khimioterapiya.

[84] Qu Y., Daley A.J., Istivan T.S., Rouch D.A., Deighton M.A. (2010). Densely adherent growth mode, rather than extracellular polymer substance matrix build-up ability, contributes to high resistance of Staphylococcus epidermidis biofilms to antibiotics. J. Antimicrob. Chemother..

[85] Seixas R., Machado J., Bernardo F., Vilela C., Oliveira M. (2014). Biofilm Formation by Salmonella Enterica Serovar 1,4, 5,12:i:- Portuguese Isolates: A Phenotypic, Genotypic, and Socio-geographic Analysis. Curr. Microbiol..

[86] Han X., Li Q., Shen L., Hu D., Qu Y. (2014). Correlation between the biofilm-forming ability, biofilm-related genes and antimicrobial resistance of Acinetobacter baumannii. Zhonghua Wei Zhong Bing Ji Jiu Yi Xue.

[87] Pozzi C., Waters E.M., Rudkin J.K., Schaeffer C.R., Lohan A.J., Tong P., Loftus B.J., Pier G.B., Fey P.D., Massey R.C. (2012). Methicillin Resistance Alters the Biofilm Phenotype and Attenuates Virulence in Staphylococcus aureus Device-Associated Infections. Plos Pathog..

[88] McCluskey J., Hinds J., Husain S., Witney A., Mitchell T.J. (2004). A two-component system that controls the expression of pneumococcal surface antigen A (PsaA) and regulates virulence and resistance to oxidative stress in Streptococcus pneumoniae. Mol. Microbiol..

[89] Coelho L.R., Souza R.R., Ferreira F.A., Guimaraes M.A., Ferreira-Carvalho B.T., Sa Figueiredo A.M. (2008). agr RNAIII divergently regulates glucose-induced biofilm formation in clinical isolates of Staphylococcus aureus. Microbiol. Sgm..

[90] Tamber S., Cheung A.L. (2009). SarZ Promotes the Expression of Virulence Factors and Represses Biofilm Formation by Modulating SarA and agr in Staphylococcus aureus. Infect. Immun..

[91] Gimza B.D., Larias M.I., Budny B.G., Shaw L.N. (2019). Mapping the Global Network of Extracellular Protease Regulation in Staphylococcus aureus. mSphere.

[92] Green E.R., Mecsas J. (2016). Bacterial secretion systems: An overview. Virulence Mech. Bact. Pathog..

[93] Costa T.R., Felisberto-Rodrigues C., Meir A., Prevost M.S., Redzej A., Trokter M., Waksman G. (2015). Secretion systems in Gram-negative bacteria: Structural and mechanistic insights. Nat. Rev. Microbiol..

[94] Rapisarda C., Fronzes R. (2018). Secretion Systems Used by Bacteria to Subvert Host Functions. Curr. Issues Mol. Biol..

[95] Thomas S., Holland I.B., Schmitt L. (2014). The type 1 secretion pathway—The hemolysin system and beyond. Biochim. Et Biophys. Acta (Bba)-Mol. Cell Res..

[96] Hentzer M., Wu H., Andersen J.B., Riedel K., Rasmussen T.B., Bagge N., Kumar N., Schembri M.A., Song Z., Kristoffersen P. (2003). Attenuation of Pseudomonas aeruginosa virulence by quorum sensing inhibitors. Embo J..

[97] Maura D., Hazan R., Kitao T., Ballok A.E., Rahme L.G. (2016). Evidence for direct control of virulence and defense gene circuits by the Pseudomonas aeruginosa quorum sensing regulator, MvfR. Sci. Rep..

[98] Teschler J.K., Zamorano-Sánchez D., Utada A.S., Warner C.J., Wong G.C., Linington R.G., Yildiz F.H. (2015). Living in the matrix: Assembly and control of Vibrio cholerae biofilms. Nat. Rev. Microbiol..

[99] Grohmann E., Christie P.J., Waksman G., Backert S. (2018). Type IV secretion in Gram-negative and Gram-positive bacteria. Mol. Microbiol..

[100] Li P., Tian M., Bao Y., Hu H., Liu J., Yin Y., Ding C., Wang S., Yu S. (2017). Brucella rough mutant induce macrophage death via activating IRE1α pathway of endoplasmic reticulum stress by enhanced T4SS secretion. Front. Cell. Infect. Microbiol..

[101] Saurav K., Bar-Shalom R., Haber M., Burgsdort I., Oliviero G., Costantino V., Morgenstern D., Steindler L. (2016). In Search of Alternative Antibiotic Drugs: Quorum-Quenching Activity in Sponges and Their Bacterial Isolates. Front. Microbiol..

[102] Ciric A.D., Petrovic J.D., Glamoclija J.M., Smiljkovic M.S., Nikolic M.M., Stojkovic D.S., Sokovic M.D. (2019). Natural products as biofilm formation antagonists and regulators of quorum sensing functions: A comprehensive review update and future trends. S. Afr. J. Bot..

[103] Dong Y.H., Wang L.H., Xu J.L., Zhang H.B., Zhang X.F., Zhang L.H. (2001). Quenching quorum-sensing-dependent bacterial infection by an N-acyl homoserine lactonase. Nature.

[104] Wu S.B., Liu J.H., Liu C.J., Yang A.D., Qiao J.J. (2009). Quorum sensing for population-level control of bacteria and potential therapeutic applications. Cell. Mol. Life Sci..

[105] Bhardwaj A.K., Vinothkumar K., Rajpara N. (2013). Bacterial quorum sensing inhibitors: Attractive alternatives for control of infectious pathogens showing multiple drug resistance. Recent Pat. Anti-Infect. Drug Discov..

[106] Han X., Lu C. (2009). Biological activity and identification of a peptide inhibitor of LuxS from Streptococcus suis serotype 2. Fems Microbiol. Lett..

[107] Schramm V.L., Gutierrez J.A., Cordovano G., Basu I., Guha C., Belbin T.J., Evans G.B., Tyler P.C., Furneaux R.H. (2008). Transition state analogues in quorum sensing and SAM recycling. Nucleic acids Symp. Ser..

[108] Storz M.P., Allegretta G., Kirsch B., Empting M., Hartmann R.W. (2014). From in vitro to in cellulo: Structure-activity relationship of (2-nitrophenyl)methanol derivatives as inhibitors of PqsD in Pseudomonas aeruginosa. Org. Biomol. Chem..

[109] Kalia M., Yadav V.K., Singh P.K., Dohare S., Sharma D., Narvi S.S., Agarwal V. (2019). Designing quorum sensing inhibitors of Pseudomonas aeruginosa utilizing FabI: An enzymic drug target from fatty acid synthesis pathway. 3 Biotech.

[110] Zhang J.W., Xuan C.G., Lu C.H., Guo S., Yu J.F., Asif M., Jiang W.J., Zhou Z.G., Luo Z.Q., Zhang L.Q. (2019). AidB, a Novel Thermostable N-Acylhomoserine Lactonase from the *Bacterium Bosea* sp. Appl. Environ. Microbiol..

[111] Zhang J., Wang J., Feng T., Du R., Tian X., Wang Y., Zhang X.-H. (2019). Heterologous Expression of the Marine-Derived Quorum Quenching Enzyme MomL Can Expand the Antibacterial Spectrum of Bacillus brevis. Mar. Drugs.

[112] Cui T.Q., Bai F.L., Sun M.T., Lv X.R., Li X.P., Zhang D.F., Du H. (2020). Lactobacillus crustorum ZHG 2-1 as novel quorum-quenching bacteria reducing virulence factors and biofilms formation of Pseudomonas aeruginosa. Lwt-Food Sci. Technol..

[113] Yu L., Li W., Zhang M., Cui Y., Chen X., Ni J., Yu L., Shang F., Xue T. (2018). Imidazole decreases the ampicillin resistance of an Escherichia coli strain isolated from a cow with mastitis by inhibiting the function of autoinducer 2. J. Dairy Sci..

[114] Roy V., Fernandes R., Tsao C.-Y., Bentley W.E. (2010). Cross species quorum quenching using a native AI-2 processing enzyme. Acs Chem. Biol..

[115] Wei Q., Bhasme P., Wang Z., Wang L., Wang S., Zeng Y., Wang Y., Ma L.Z., Li Y. (2020). Chinese medicinal herb extract inhibits PQS-mediated quorum sensing system in Pseudomonas aeruginosa. J. Ethnopharmacol..

[116] Truchado P., Gimenez-Bastida J.-A., Larrosa M., Castro-Ibanez I., Carlos Espin J., Tomas-Barberan F.A., Teresa Garcia-Conesa M., Allende A. (2012). Inhibition of Quorum Sensing (QS) in Yersinia enterocolitica by an Orange Extract Rich in Glycosylated Flavanones. J. Agric. Food Chem..

[117] Ryu E.-J., Sim J., Sim J., Lee J., Choi B.-K. (2016). D-Galactose as an autoinducer 2 inhibitor to control the biofilm formation of periodontopathogens. J. Microbiol..

[118] Sun B., Zhang M. (2016). Analysis of the antibacterial effect of an Edwardsiella tarda LuxS inhibitor. SpringerPlus.

[119] Halbedl S., Kratzer M.C., Rahm K., Crosta N., Masters K.S., Zippert J., Brase S., Gradl D. (2013). Synthesis of novel inhibitors blocking Wnt signaling downstream of beta-Catenin. Febs Lett..

[120] Takayama H., Jia Z.J., Kremer L., Bauer J.O., Strohmann C., Ziegler S., Antonchick A.P., Waldmann H. (2013). Discovery of Inhibitors of the Wnt and Hedgehog Signaling Pathways through the Catalytic Enantioselective Synthesis of an Iridoid-Inspired Compound Collection. Angew. Chem. Int. Ed..

[121] Blocher R., Ramirez A.R., Castro-Escarpulli G., Curiel-Quesada E., Reyes-Arellano A. (2018). Design, Synthesis, and Evaluation of Alkyl-Quinoxalin-2(1H)-One Derivatives as Anti-Quorum Sensing Molecules, Inhibiting Biofilm Formation in Aeromonas caviae Sch3. Molecules.

[122] Bortolotti D., Trapella C., Bragonzi A., Marchetti P., Zanirato V., Alogna A., Gentili V., Cervellati C., Valacchi G., Sicolo M. (2019). Conjugation of LasR Quorum-Sensing Inhibitors with Ciprofloxacin Decreases the Antibiotic Tolerance of P-aeruginosa Clinical Strains. J. Chem..

[123] Pena R.T., Blasco L., Ambroa A., Gonzalez-Pedrajo B., Fernandez-Garcia L., Lopez M., Bleriot I., Bou G., Garcia-Contreras R., Wood T.K. (2019). Relationship Between Quorum Sensing and Secretion Systems. Front. Microbiol..

[124] Grandclement C., Tannieres M., Morera S., Dessaux Y., Faure D. (2016). Quorum quenching: Role in nature and applied developments. Fems Microbiol. Rev..

[125] Liu J.Y., Zhou R., Li L., Peters B.M., Li B., Lin C.W., Chuang T.L., Chen D.Q., Zhao X.H., Xiong Z.Y. (2017). Viable but non-culturable state and toxin gene expression of enterohemorrhagic Escherichia coli 0157 under cryopreservation. Res. Microbiol..

[126] Zhao X., Lin C.W., Wang J., Oh D.H. (2014). Advances in Rapid Detection Methods for Foodborne Pathogens. J. Microbiol. Biotechnol..

[127] Metz B., Jiskoot W., Hennink W.E., Crommelin D.J., Kersten G.F. (2003). Physicochemical and immunochemical techniques predict the quality of diphtheria toxoid vaccines. Vaccine.

[128] Quan Y., Meng F., Ma X., Song X., Liu X., Gao W., Dang Y., Meng Y., Cao M., Song C. (2017). Regulation of bacteria population behaviors by AI-2 “consumer cells” and “supplier cells”. Bmc Microbiol..

[129] Zhu J., Hixon M.S., Globisch D., Kaufmann G.F., Janda K.D. (2013). Mechanistic insights into the LsrK kinase required for autoinducer-2 quorum sensing activation. J. Am. Chem. Soc..

[130] Xavier K.B., Miller S.T., Lu W., Kim J.H., Rabinowitz J., Pelczer I., Semmelhack M.F., Bassler B.L. (2007). Phosphorylation and processing of the quorum-sensing molecule autoinducer-2 in enteric bacteria. Acs Chem. Biol..

[131] Stotani S., Gatta V., Medarametla P., Padmanaban M., Karawajczyk A., Giordanetto F., Tammela P.i., Laitinen T., Poso A., Tzalis D. (2019). DPD-inspired discovery of novel LsrK kinase inhibitors: An opportunity to fight antimicrobial resistance. J. Med. Chem..

[132] Neiditch M.B., Federle M.J., Pompeani A.J., Kelly R.C., Swem D.L., Jeffrey P.D., Bassler B.L., Hughson F.M. (2006). Ligand-induced asymmetry in histidine sensor kinase complex regulates quorum sensing. Cell.

[133] Shao C., Shang W., Yang Z., Sun Z., Li Y., Guo J., Wang X., Zou D., Wang S., Lei H. (2012). LuxS-dependent AI-2 regulates versatile functions in Enterococcus faecalis V583. J. Proteome Res..

[134] Xiao Y., Yaohari H., Zhou Z., Sze C.C., Stuckey D.C. (2019). Autoinducer-2-mediated quorum sensing partially regulates the toxic shock response of anaerobic digestion. Water Res..

[135] Stevens A.M., Queneau Y., Soulere L., von Bodman S., Doutheau A. (2011). Mechanisms and Synthetic Modulators of AHL-Dependent Gene Regulation. Chem. Rev..

[136] Smith K.M., Bu Y., Suga H. (2003). Library screening for synthetic agonists and antagonists of a Pseudomonas aeruginosa autoinducer. Chem. Biol..

